# The first assessment of marine litter on somalian coast: The case of Liido Beach, mogadishu

**DOI:** 10.1016/j.heliyon.2024.e26593

**Published:** 2024-02-16

**Authors:** Hassan O. Hassan, Emuobonuvie G. Ayeta, Abdisatar A. Ibrahim, Mohamed F. Omar, Suweyda M. Abdi, Youssouf K. Houmed, Abdulrahman M. Dirie, Charles A. Faseyi

**Affiliations:** a Somali Disaster Management Agency, Somalia; b Centre for Coastal Management, Africa Centre of Excellence in Coastal Resilience, University of Cape Coast, Ghana; c Department of Fisheries and Aquatic Sciences, University of Cape Coast, Ghana; d Department of Engineering, Mogadishu University, Somalia; e Greenlight Association, Mogadishu, Somalia; f Somali Youth Empowerment Programme, Mogadishu, Somalia; g Green Climate Fund Readiness Project, Global Water Partnerships Africa, Somalia

**Keywords:** Marine debris, Plastic pollution, Litter density, Clean coast index

## Abstract

This paper presents the first assessment of marine litter in the Mogadishu coastal area of Somalia.

Samples were collected monthly using 100 m × 40 m transect and classified following OSPAR Marine Litter Survey Guide while litter sources were identified using Ocean Conservancy Marine Debris Index. The results showed a total of 119873 items consisting of plastics (89.47%), clothing items (7.53%), and others (3.00%) recovered from Liido Beach. Litter density ranged from 2.19 items/m^2^ to 14.18 items/m^2^ with a mean of 6.25 items/m^2^ and Clean Coast Index (CCI) suggesting that Liido Beach is extremely dirty (>20 items/m^2^). In addition, the primary sources of marine litter at the beach are local recreational and shoreline activities (54.12%), and dumping (36.61%). The dominance of plastic litter on the beach poses potential threats to marine biodiversity in the Somalia coastal area and the West Indian Ocean. It is recommended that effective strategies and solutions to mitigate litter on the beach and other coastal areas in Somalia should be developed and compensated with public education and awareness campaigns across the country.

## 1 Introduction

Coastal urban centres are subjected to high levels of human activities, which produce a lot of litter or waste with little or no capacity to manage effectively [[1], [2], [3], [4]]. As a result, large volumes of waste are deposited in open areas or natural drainage systems, eventually ending up on the coast. Marine litter, also known as marine debris is defined as any man-made object discarded, disposed of, or abandoned, that enters the coastal or marine environment [[5], [6], [7], [8], [9], [10]]. These include household items, industrial products, as well as lost or discarded fishing gear, recreational equipment, and plastic pieces [8,11]. Marine litter can pose a risk to human health, environmental integrity, social and economic well-being, as well as livelihoods [6,[12], [13], [14], [15], [16]]. They have been described as one of the most common pollution of concerns plaguing the world's oceans and waterways [[17], [18], [19], [20]]. Marine litter occurs in all regions of the world regardless of socio-cultural and economic status; however, gaps in policies and management strategies exist [15,[21], [22], [23]].

According to UNEP [24], land-based sources account for about 80% of marine litter. Coastal towns are estimated to generate 24.6% of municipal solid waste in Sub-Saharan Africa, with the majority of it being non-recoverable, and most of it being dumped into water bodies [24]. Coastal zones act as pollution sinks that prolong the pathways of marine pollutants resulting in serious water quality issues, coastal eutrophication, and a decline in wetland areas, as well as beach closures caused by faeces, plastics, and other pollutants [6,25,26]. Marine pollution endangers biodiversity and poses considerable economic and health risks to fisheries and coastal communities [14,[27], [28], [29], [30], [31], [32], [33]].

Somali coast is bordered by Western Indian Ocean (WIO) which is home to a range of productive marine ecosystems that provide food, livelihoods, and natural beauty. The Western Indian Ocean is rich in a diverse range of species and high levels of endemism and is one of the richest oceanic locations on the planet [24,[45], [46], [47], [48], [49]]. It is, however, vulnerable to the effects of marine pollution caused by human activities.

Somalia has the second longest coastline in Africa (3025 km). Its shoreline consists of sand beaches, dunes, cliffs, fringing coral reefs, and patchy reefs. Its economy relies heavily on natural resources, including agriculture, livestock, and fishing, with the latter presenting a promising avenue for revenue generation [50]. Although, there are studies in Africa on marine litter (Table 1), but there is currently no information from Somalia and the research in this area is in its infancy. Therefore, this paper aims to provide the first assessment of marine litter in Somalia by determining the composition and abundance, as well as identifying the sources at Liido Beach in Mogadishu.

**Table 1:** Title and findings of some marine litter research in Africa.

Title	Country	Key Findings	Reference
Composition and potential origin of marine debris stranded in the Western Indian Ocean on remote Alphonse Island, Seychelles	Seychelles	The study discovered 4743 items weighing 142 kg, typically plastics, indicating the prevalence of plastic debris as a global marine pollutant. The debris, mostly from sources on land, was traced back to Southeast Asia and Somalia. The paper concludes that poor waste management practices resulted in the debris entering the Western Indian Ocean.	[27]
Spatial and temporal variations in beach litter on the Transkei coast of South Africa	South Africa	Reported that litter counts and weights varied significantly between beaches, with plastics being the most commonly found type of litter. In addition, tourist beaches had the widest variety of litter types.	[34]
Temporal trends of marine litter in a tropical recreational beach: A case study of Mkomani beach, Kenya.	Kenya	The study found branded litter on the Mkomani beach which were mainly consisted of foam and plastic fragments. Interestingly, 66.9% of the branded litter was of Kenyan origin while 78.4% of the litter was from food product packaging, especially Polyethylene Terephthalate (PET) bottles. The beach was labelled as “extremely dirty."	[23]
Baseline meso-litter pollution in selected coastal beaches of Kenya: Where do we concentrate our intervention efforts?	Kenya	Twenty-three (23) beaches were surveyed and recorded plastics as the most abundant litter, with close urban areas having higher litter categories.	[9]
Marine litter in the central Atlantic coast of Morocco	Morocco	It revealed plastics made up 79% of the litter items collected on the coast while metal was the second most common material found. It identified areas with high levels of pollution, with tourism and recreational activities being the primary sources of marine litter. Four solutions were suggested to address this issue: cleaning, prevention, mitigation, and encouraging behaviour change.	[35]
Contribution to the assessment of marine litter in the North Moroccan Atlantic	Morocco	The survey carried out a bottom trawling campaign to collect data at 60 stations. A total of 13.5 kg of waste was recorded, with 81% being plastics. The primary source of the waste is associated with fishing activities.	[36]
The impacts of COVID-19 pandemic on marine litter pollution along the Kenyan Coast: A synthesis after 100 days following the first reported case in Kenya	Kenya	COVID-19 was found to contribute to marine litter deposition and accounted for 16.5% of the sources of the litter surveyed.	[37]
Influence of tourist pressure on beach Litter and microbial quality - Case study of two beach resorts in Ghana	Ghana	Plastics were reported to be the most common type of litter collected during the festive season, with visitor pressure having a direct impact on the quantity of litter present.	[38]
Land or sea? What bottles tell us about the origins of beach litter in Kenya	Kenya	According to the study, PET drink bottles that are locally manufactured are the primary source of street litter and urban beach pollution in Kenya. Meanwhile, High-Density Polyethylene (HDPE) bottles, which mostly come from Indonesia, are more common on beaches. Foreign PET bottles mainly come from Indian Ocean-island states.	[39]
Message in a bottle: Assessing the sources and origins of beach litter to tackle marine pollution	South Africa	Thirty-two (32) sites were surveyed, finding plastics as the most common litter. South African bottles dominated street litter, while foreign-manufactured bottles were prevalent at some beaches. The majority of HDPE recovered was from Indonesia.	[40]
The effect of fine-scale sampling frequency on estimates of beach litter accumulation	South Africa	The survey found that weekly samples did not consistently show less variation than daily samples. This suggests that less frequent samples only partially integrate short-term fluctuations in litter dynamics.	[41]
Low densities of drifting litter in the African sector of the Southern Ocean	South Africa	Reported 52 small litter items, less than 1 cm in diameter, with 96% of them being made of plastics. It also confirmed that the Southern Ocean is the least polluted in terms of drifting debris. It was suggested that most of the debris comes from local sources.	[42]
Consistent patterns of debris on South African beaches indicate that industrial pellets and other mesoplastic items mostly derive from local sources	South Africa	According to the survey, 95% of litter items by mass and 99% of litter items by number are made of plastics. Plastic objects that are most commonly found are industrial pellets. Additionally, the study discovered a high association between other plastic products and industrial pellets, suggesting that similar variables affect the dispersion of secondary mesoplastics and pellets.	[6]
Spatiotemporal variations in marine litter along the Gulf of Guinea coastline, Araromi seaside, Nigeria	Nigeria	A survey of 20 sites conducted, revealing 29029 litter items, with plastic items being the most common. The location was found to be highly dirty during the wet season and dirty during the dry season, with numerous dangerous objects scattered throughout.	[43]
Suspended Marine Litter in Akwa Ibom State, Nigeria: A Case Study of Cross River, QUA Iboe River and Jaja Creek	Nigeria	It revealed that the navigable canals in the area are obstructed by suspended debris such as plastics, nylons, foils, and cans. The highest weight was found at 90220 kg/km^2^ at Akpam Nfrugam and 199820 kg/km^2^ at Jaja Creek.	[44]

## 2 Materials and Methods

### 2.1 Study Site

**Fig. 1:**
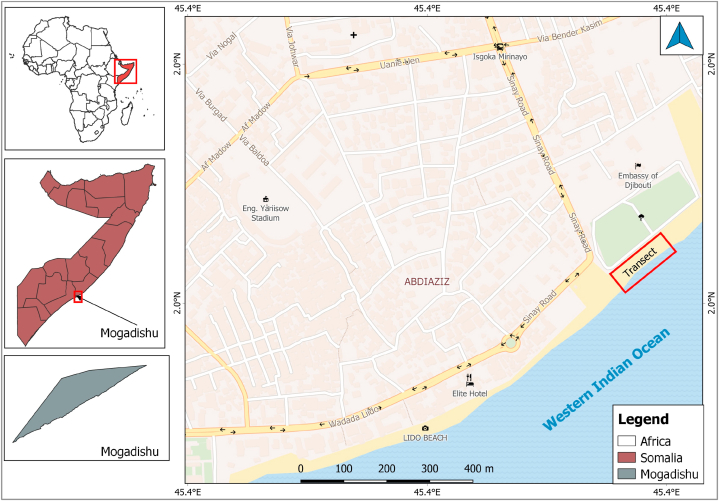
Map of Liido Beach showing the sampling location parallel to Western Indian Ocean (100 m × 40 m transect in the red box above the map Legend).

Mogadishu is the largest and the capital city of Somalia (Fig. 1). The city is situated in the Benadir region which is made up of seventeen districts. Mogadishu has a population size of 2.5 million [51], and is the second-fastest growing globally, with an annual growth rate of 4%. About 23% of this population are Internally Displaced People (IDP) who live in informal settlements, making access to basic sanitation and waste disposal facilities a problem in the city.

Somalia has bimodal rainfall patterns; a minor wet season (October to November), a major wet season (April to June), and two dry seasons; a minor dry season (July to September), a major dry season (December to March) that are influenced by Inter Tropical Convergence Zone (ITCZ) according to Muchiri [52].

The climate of Mogadishu is governed by the monsoon winds and oceanic current systems with a mean temperature of 27 °C [51]. During the period of the southwest monsoon (April to October) and eastward surface current, the north-east monsoon wind prevails during the northeast monsoon period (November to March) [52].

Mogadishu's coastal ecosystems and habitats include wide sandy beaches with rocky outcrops and a coastal plain. Liido Beach is 1.5 km long, and 500 m from the beach to the sea with a fringing reef and huge algae ecosystem which supports a developing artisanal reef fishery. The beach is an important tourist location in Mogadishu and it was selected based on the UNEP criteria for beach selection for marine litter assessment [26].

### 2.2 Data Collection

Data collection was done between November 2021 and July 2022. Six months of marine litter data surveys (one survey per month) were collected every last Friday using a 100 m × 40 m transect along the coastline parallel to the Ocean (Western Indian Ocean) to assess the composition and distribution in the Liido area. Samples for the dry season were collected in November, January and February while that the rainy season were collected in May, June and July. However, the nature of the beach made it difficult to adopt OSPAR protocol fully, therefore, the protocolof marine beach debris monitoring [53] was modified and a transect of 100 m × 40 m parallel to the sea was used. Marine litter within the transect were collected, sorted into categories using the OSPAR Marine Litter Survey list and counted.

### 2.3 Data Analysis

The data collected from the surveys were subjected to descriptive statistics (mean and range) and percentage litter compositions were estimated. To determine the abundance of litter items per unit area and relative severity of the litter pollution compared to other beaches, Litter Density (LD) and Clean Coast Index (CCI) were estimated using the formula by Ma et al. [54] and Rangel-Buitrago et al. [55] respectively.

1 $$\text{Litter}\;\text{Density}\;\left(\text{LD}\right)=\frac{N}{\left(W\;X\;L\right)}$$

where LD is the density of litter items/m^2^, N is the number of litter items recorded; W and L are the Width and Length of the sampling unit (transect), respectively.

2 CleanCoastIndex(CCI)=LDXK

where LD is the density of litter items/m^2^ and K is a constant that equals 20.

Clean Coast Index (CCI) values from 0 to 2 indicate very clean, 2–5 clean, 5–10 moderately clean, 10–20 dirty, and >20 extremely dirty. To identify the sources of marine litter on the beach, Ocean Conservancy Marine Debris Index [56] was employed.

## 3 Results

**Fig. 2:**
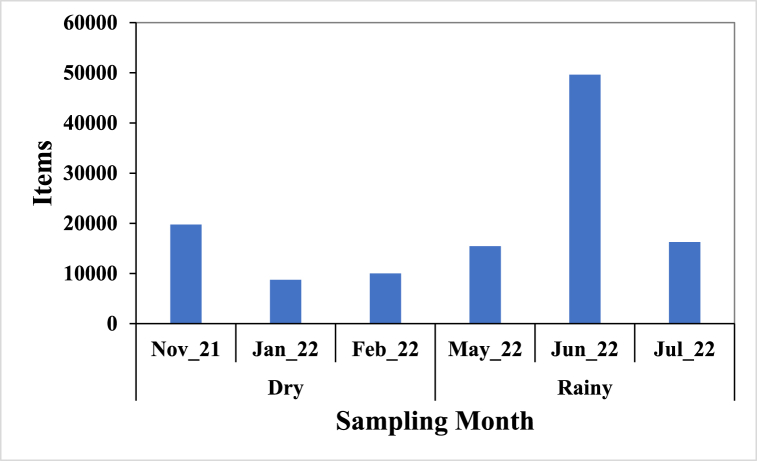
Number of litter items from Liido Beach.

Fig. 2 shows the monthly count of litter items collected and in the order June > November > July > May > February > January with a total of 119873 litter items during the six-month surveys.

**Fig. 3:**
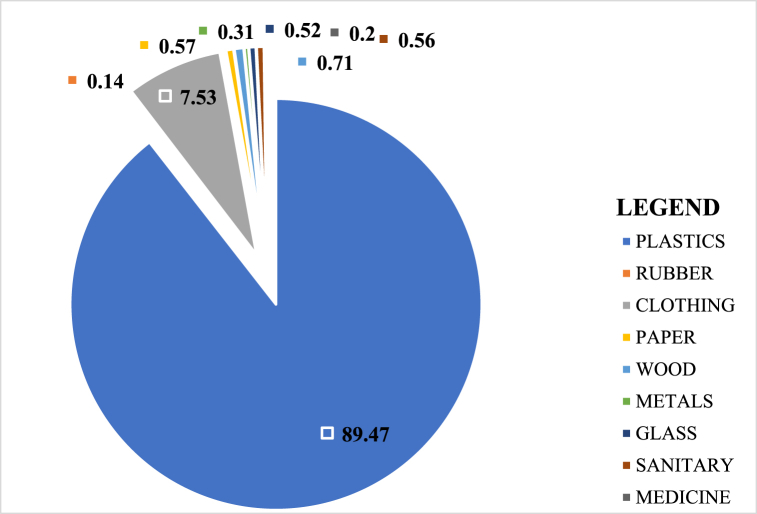
Litter percentage composition on Liido Beach.

Fig. 3 presents the percentage composition of the litter collected for the entire sampling period. The results showed that 89.47% of the litter found on Liido Beach were plastics. This was followed by clothing items which accounted for 7.53% of the total litter. Litter such as rubber, glass, sanitary, wood, glass, metals, and medicals accounted for a very small percentage (3.00%) of the total litter recorded, while faeces and ceramics were not found on the beach throughout the entire period.

**Fig. 4:**
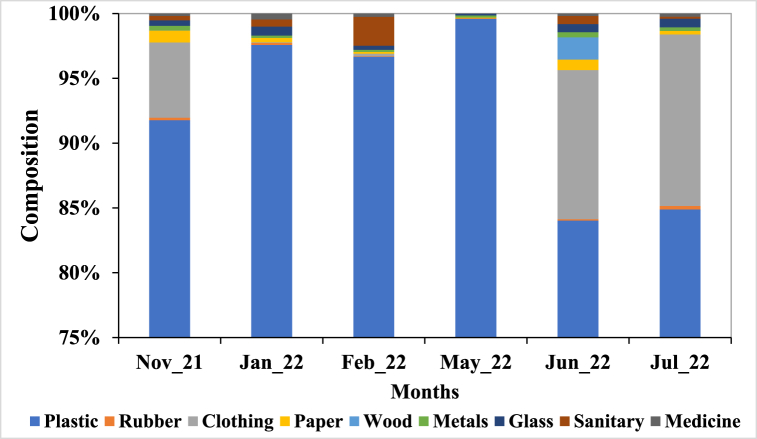
Percentage composition of litter on Liido Beach.

The monthly percentage composition of different categories or groups of litter is presented in Fig. 4. Plastic litter had the highest percentage composition across the month (November: 91.78%, January: 97.58%, February: 96.67%, May: 99.60%, June: 84.02%, and July: 84.88%) and followed by clothing items (November: 5.81%, June: 11.50%, and July: 13.22%).

**Fig. 5:**
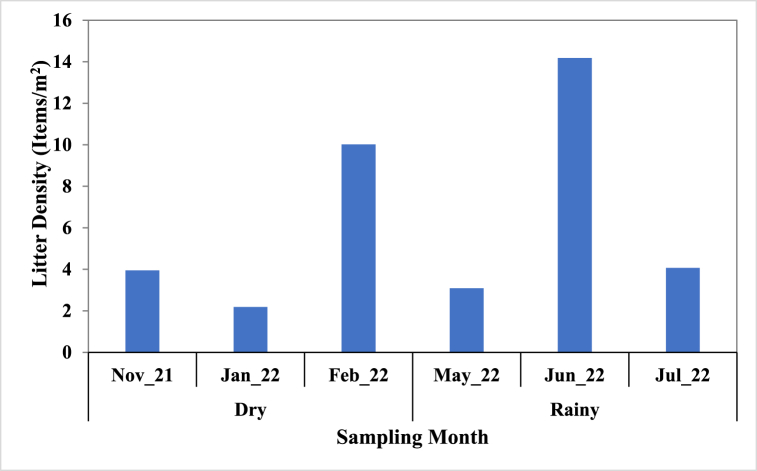
Litter density on Liido Beach.

Monthly litter density is shown in Fig. 5, June (14.18 items/m^2^) > February (10.02 items/m^2^) > July (4.07 items/m^2^) > November (3.95 items/m^2^) > May (3.09 items/m^2^) > January (2.19 items/m^2^). In overall for the period, the litter density ranged from 2.19 to 14.18 items/m^2^ with a mean of 6.25 items/m^2^.

The Clean Coast Index (CCI), representing the beach's cleanliness on a monthly basis, exhibited a mean value of 124.97 items/m^2^. June recorded the highest CCI at 283.57 items/m^2^, while January depicted the lowest at 43.72 items/m^2^. Other notable monthly CCIs were November at 79.08 items/m^2^, February at 200.32 items/m^2^, May at 61.78 items/m^2^, and July at 81.37 items/m^2^.

**Fig. 6:**
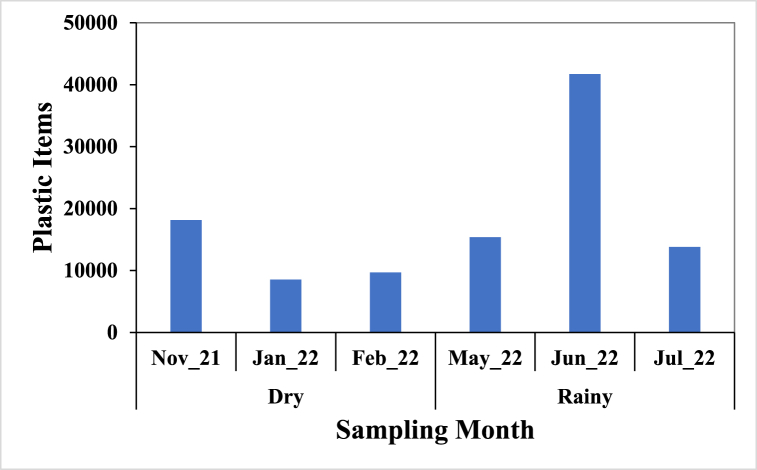
Number of plastic items recovered from Liido Beach.

**Fig. 7:**
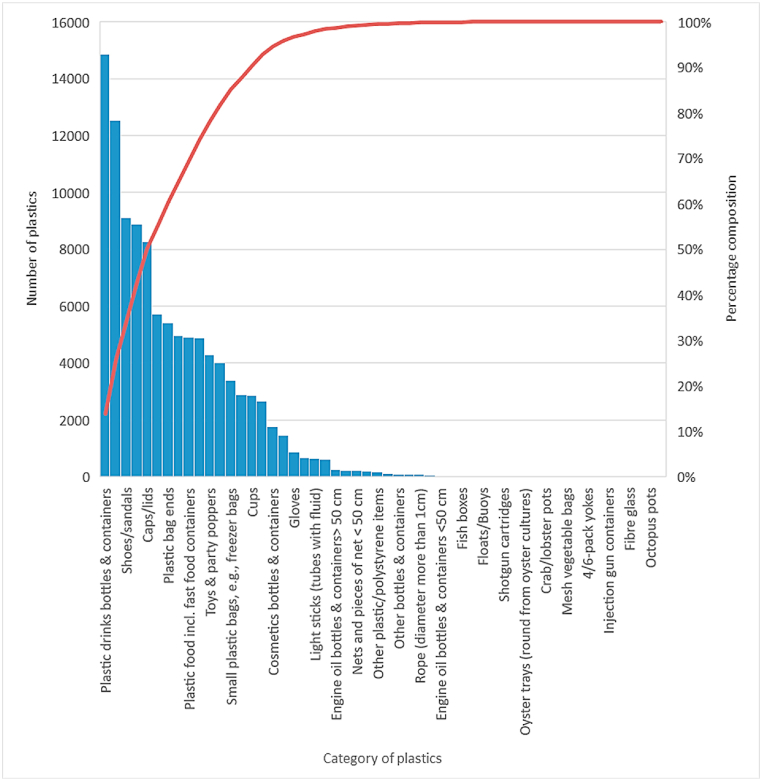
Composition of plastic items on Liido Beach.

Fig. 6, Fig. 7 show the number and composition of plastic items that dominated the litter and a total of 107249 plastic items were recovered, belonging to 40 of the 54 categories of plastics described by OSPAR [53]. Of the 40 categories found, 12 categories accounted for 81.90%. These included plastic bottles and containers (13.90%), shopping bags (11.70%), shoes/sandals (8.50%), crisp/sweet packets and lolly sticks (8.30%), and caps and lids (7.70%), plastic/polystyrene pieces of 0–2.5 cm (5.30%), plastic bag ends (5.10%), foam sponges (4.60%), food containers including fast food containers (4.60%), cutlery/trays/straws (4.50%), toys and party poppers (4.00%), plastic cleaner bottles and containers (3.70%). Most of the plastics in these 12 categories are materials used in packaging.

The potential sources of the plastics found on the beach are shown in Table 2. Shoreline and recreational activities generated the highest number of plastics in the area. Liido Beach is an important tourist attraction centre in Mogadishu, and it is used by both local and foreign tourists for several recreational activities. The shoreline and recreational activities category had the highest percentage of plastic litter, accounting for 54.12% of the total number of plastics found.

**Table 2:** Suspected sources and types of plastics on Liido Beach.

Source	Category of plastics found	Number	Percentage (%)
**Shoreline and recreational activities**	Caps/lids	8269	7.71
	Plastic drinks bottles & containers	14860	13.86
	Crisp/sweet packets and lolly sticks	8878	8.28
	Toys & party poppers	4292	4.00
	Cups	2861	2.67
	Cutlery/trays/straws	4864	4.54
	Plastic food incl. fast food containers	4907	4.58
	Shoes/sandals	9114	8.50
	**Total**	**58045**	**54.12**
**Dumping**	Bags (shopping)	12529	11.68
	Small plastic bags, e.g., freezer bags	3392	3.16
	Plastic bag ends	5421	5.05
	Plastic cleaner bottles & containers	4015	3.74
	Crates	54	0.05
	Car parts	24	0.02
	Pens	1451	1.35
	Combs/hair brushes	5	0.00
	Fertiliser/animal feed bags	80	0.07
	Gloves	853	0.80
	Hard hats	12	0.01
	Shotgun cartridges	9	0.01
	Plastic/polystyrene pieces 0–2.5 cm	5729	5.34
	Plastic/polystyrene pieces 2.5 cm > < 50 cm	2657	2.48
	Plastic/polystyrene pieces >50 cm	2881	2.69
	Other plastic/polystyrene items	157	0.15
	**Total**	**39269**	**36.61**
**Ocean/waterways activities**	Rope (diameter more than 1 cm)	71	0.07
	String and cord (diameter leases than 1 cm)	202	0.19
	Nets and pieces of net <50 cm	233	0.22
	Nets and pieces of net >50 cm	104	0.10
	Fish boxes	27	0.03
	Fishing line (angling)	678	0.63
	Light sticks (tubes with fluid)	647	0.60
	Floats/Buoys	13	0.01
	Strapping bands	609	0.57
	Engine oil bottles & containers <50 cm	34	0.03
	Engine oil bottles & containers> 50 cm	241	0.22
	Jerry cans (square plastic containers with handles)	32	0.03
	Foam sponge	4959	4.62
	**Total**	**7850**	**7.32**
**Medical and sanitary waste**	Cosmetics bottles & containers	1771	1.65
	Other bottles & containers	81	0.08
	**Total**	**1852**	**1.73**
**Smoking-related**	Cigarette lighters	233	0.22
	**Total**	**233**	**0.22**
	**TOTAL NUMBER OF PLASTICS**	**107249**	

## 4 Discussion

A total number of 119873 litter items collected from Liido Beach is an indication of environmental pollution from anthropogenic activities. The number recorded was higher than the 4952 items jointly recorded in Korle and La Beaches (Ghana) by Tsagbey et al. [38], and a mean of 32577 reported by Kienitz [57] from six bays in Hornstrandir (Iceland). Also, a total of 445 items were reported in Guafo Island (Chile) from 2013 to 2017 [58] while 4743 items were reported by Duhec et al. [27] in Alphonse Island (Seychelles) and 1438 items were reported in Car Nicobar Beach [59]. This high number of litter items on the beach (Liido Beach) shows the level of marine pollution in the country as compared to some studies around the world. Forty individual items were recorded in this survey which was slightly more than the 32 individual items by Tsagbey et al. [38] but lower when compared to 104 individual items reported by Kienitz [57]. There were temporal variations in the number of litter items, the highest number of litter items were recorded in June (49625 items) which could result from runoff transporting litter upland to the beach while the least was recorded in January which is the pick of the dry season in Somalia. A similar trend was reported by Tsagbey et al. [38] in Ghana. The differences in litter items across months indicate distinct variations in litter accumulation, emphasising the seasonal dynamics influencing debris deposition along the Somalian coast.

In overall, the litter density ranged from 2.19 items/m^2^ to 14.18 items/m^2^ with a mean of 6.25 items/m^2^. These results are higher than the value obtained at the Columbian Central Caribbean Beaches (5.11 items/m^2^), the Zhoushan Island, and the Adriatic and Ionian Beaches (0.67 items/m^2^) by Rangel-Buitrago et al. [55], Ma et al. [54], and Vlachogianni et al. [60] respectively. Perez-Venegas et al. [58] recorded a litter density of 0.001 items/m^2^ on Guafo Island (Chile) while Duhec et al. [27] reported 4.7 items/m^2^ on Alphonse Island (Seychelles), and 3.3 items/100 m^2^ was recorded by Melli et al. [61].

According to CCI scale; values from 0 to 2 indicate very clean beaches, 2–5 clean, 5–10 moderately clean, 10–20 dirty, and >20 extremely dirty. Therefore, Liido Beach is extremely dirty as the CCI values recorded were greater than 20, with a mean value of 124.97 items/m^2^ in all the sampling months. Similar extremely dirty coasts were reported by Akarsu et al. [62] on a Turkish beach, and Mishra et al. [63] on a Pan-India beach while Mugilarasan et al. [4] reported moderately clean CCI in the southeastern Arabian Sea coast (India).

Liido Beach was dominated by plastics which accounted for 89.47% of the total items recovered. The percentage composition of plastic items recorded was higher than those reported by Tsagbey et al. [38] who reported 66% and 50% plastic items in Korle and La Beaches respectively in Ghana. Kienitz [57] recorded 95.4% of plastic litter from Hornstrandir (Iceland) while Duhec et al. [27] reported 43.43% plastic litter in Alphonse Island, and Kiruba-Sankar et al. [59] recorded 83.72% in Car Nicobar Beach. It is not surprising as plastics have been noted to be the most abundant marine litter worldwide [55,64,65]. However, plastics are non-biodegradable, persist in the environment for a very long time, and have a very deleterious effect on marine organisms. Dominant plastic litter reported on the Somalian coast is similar to the findings of Rangel-Buitrago et al. [55] who found 43 categories of plastics on the beaches along the Caribbean coast. The most frequently found plastic items in this category were caps/lids, plastic drinks bottles and containers, crisp/sweet packets and lolly sticks, and toys and party poppers. This suggests that these items are commonly used and disposed of by beachgoers, leading to a high abundance of plastic litter on the beach. Furthermore, the high number of plastic waste from the shoreline and recreational activities (54.12%) shows the poor waste management practices of the coastal dwellers along the Somalian coast and beach users. This poor attitude may be due to low education on the effects of marine waste and the absence of waste bins along Liido Beach. Ololaha Nadaafada Xeebta Liido Beach cleaning project has been actively carrying out this form of education at the beach during its weekly cleanup activities. However, more efforts are needed from both individuals and cooperate groups to ensure that the Mogadishu coastline is free of litter. Dumping also represented a significant source of plastics in the area, as about 36.61% of the plastic waste found on the beach was due to dumping. Household wastes such as shopping bags formed the largest proportion of the dumped materials. Single-use shopping bags are a major source of land-based marine litter [66]. Several methods including bans, fees, taxes, and awareness have been used in different jurisdictions to control the use of single-use shopping bags [[66], [67], [68]], but these have met with several obstacles. Despite its ban on plastic shopping bags, the coastline of Somalia still suffers from pollution from plastic bags. The presence of plastic litter in Liido Beach has potential threats to marine biodiversity in Somalian coast and West Indian Ocean.

## 5 Conclusion

The first comprehensive assessment of marine litter composition and sources on Liido Beach, Mogadishu, Somalia was presented. The paper described an environmental challenge of a beach battling a significant marine litter problem. The highest litter density was recorded during June, which is the rainy season, at a rate of 14.18 items/m^2^. This depicts a beach littered with debris, diminishing its aesthetic appeal and posing ecological threats that vary seasonally. Dominating the litter composition were plastics (89.47%) followed by clothing items (7.53%) confirming plastics as a ubiquitous contaminant plaguing oceans worldwide. Fishing gear and glass fragments also emerged as major contributors, highlighting the diverse nature of the pollution on Liido Beach. Local recreational activities, shoreline misuse, and waste dumping were pinpointed as the primary culprits behind the beach's litter pollution. This emphasises the need for targeted interventions within the community. Therefore, these findings provided the baseline data for crucial strategies to combat marine litter not just at Liido Beach but across Somalia's coast. Public education and awareness campaigns are essential to foster responsible waste disposal practices. Furthermore, implementing effective litter control measures, including regular clean-up drives and improved waste management infrastructure, is crucial.

## Funding

There was no funding for this study.

## Ethics declarations

This study did not involve human subjects, so ethical clearance was not needed.

## Data availability statement

The data associated with this study has not been deposited into any public repository and can be made available on request.

## CRediT authorship contribution statement

**Hassan O. Hassan:** Writing – review & editing, Writing – original draft, Visualization, Formal analysis, Conceptualization. **Emuobonuvie G. Ayeta:** Writing – review & editing, Writing – original draft, Visualization, Methodology, Formal analysis. **Abdisatar A. Ibrahim:** Writing – original draft, Formal analysis, Conceptualization. **Mohamed F. Omar:** Writing – original draft, Formal analysis, Conceptualization. **Suweyda M. Abdi:** Writing – original draft, Formal analysis, Conceptualization. **Youssouf K. Houmed:** Writing – original draft, Formal analysis, Conceptualization. **Abdulrahman M. Dirie:** Writing – original draft, Formal analysis, Conceptualization. **Charles A. Faseyi:** Writing – review & editing, Writing – original draft, Visualization, Methodology, Formal analysis.

## Declaration of competing interest

The authors declare that they have no known competing financial interests or personal relationships that could have appeared to influence the work reported in this paper.
